# Soluble Fibre Meal Challenge Reduces Airway Inflammation and Expression of GPR43 and GPR41 in Asthma

**DOI:** 10.3390/nu9010057

**Published:** 2017-01-10

**Authors:** Isabel Halnes, Katherine J. Baines, Bronwyn S. Berthon, Lesley K. MacDonald-Wicks, Peter G. Gibson, Lisa G. Wood

**Affiliations:** 1 Centre for Healthy Lungs, Hunter Medical Research Institute, University of Newcastle, Callaghan, NSW 2308, Australia; isabel_h90@hotmail.com (I.H.); Katherine.baines@newcastle.edu.au (K.J.B.); bronwyn.berthon@newcastle.edu.au (B.S.B.); peter.gibson@newcastle.edu.au (P.G.G.); 2 School of Health Sciences, University of Newcastle, Callaghan, NSW 2305, Australia; lesley.wicks@newcastle.edu.au

**Keywords:** asthma, prebiotics, G-protein coupled receptor, inflammation, short chain fatty acids

## Abstract

Short chain fatty acids (SCFAs) are produced following the fermentation of soluble fibre by gut bacteria. In animal models, both dietary fibre and SCFAs have demonstrated anti-inflammatory effects via the activation of free fatty acid receptors, such as G protein-coupled receptor 41 and 43 (GPR41 and GPR43). This pilot study examined the acute effect of a single dose of soluble fibre on airway inflammation—including changes in gene expression of free fatty acid receptors—in asthma. Adults with stable asthma consumed a soluble fibre meal (*n* = 17) containing 3.5 g inulin and probiotics, or a control meal (*n* = 12) of simple carbohydrates. Exhaled nitric oxide (eNO) was measured and induced sputum was collected at 0 and 4 h for differential cell counts, measurement of interleukin-8 (IL-8) protein concentration, and GPR41 and GPR43 gene expression. At 4 h after meal consumption, airway inflammation biomarkers, including sputum total cell count, neutrophils, macrophages, lymphocytes, sputum IL-8, and eNO significantly decreased compared to baseline in the soluble fibre group only. This corresponded with upregulated GPR41 and GPR43 sputum gene expression and improved lung function in the soluble fibre group alone. Soluble fibre has acute anti-inflammatory effects in asthmatic airways. Long-term effects of soluble fibre as an anti-inflammatory therapy in asthma warrants further investigation.

## 1. Introduction

Asthma is a chronic inflammatory disease of the airways, affecting approximately 300 million people worldwide [1]. In Australia, over two million people are affected [2]. Glucocorticoids are the mainstay of asthma management [3]. However, treatment can predispose individuals to long-term side effects (e.g., osteoporosis, hypertension, insulin resistance, neuropsychiatric effects), and some patients respond poorly [3]. Thus, new ways to treat inflammation in asthma are urgently needed.

While asthma has a significant genetic component, environmental factors also play a role. Epidemiological studies report increased asthma risk with consumption of westernized diets [4,5,6,7], which are often high in fat and processed foods, and low in fibre. Dietary fibres are complex carbohydrates found in plant-based foods and are classified based on their solubility in water [8]. Insoluble fibres (e.g., cellulose) are biologically inert substances that assist in the alleviation of constipation by promoting bowel movements [8]. Soluble fibres such as oligosaccharides and fructans (e.g., inulin) act as substrates for fermentation by intestinal microbes [9]. Partial digestion of soluble fibre produces short chain fatty acids (SCFAs), primarily acetate, propionate and butyrate [9]. Butyrate is the preferred fuel source for colonocytes, propionate is mainly metabolized by the liver, while acetate is the main SCFA to enter the circulation where it can act on immune cells and peripheral tissues and potentially elicit anti-inflammatory effects [9,10]. 

Recently, we reported that fibre intake was inversely associated with lung function and eosinophilic airway inflammation [11,12]. One mechanism potentially linking dietary fibre intake and inflammatory responses, involves the activation of the free fatty acid receptors, G protein-coupled receptor 41 and 43 (GPR41 and GPR43). These are cell surface receptors that are activated by SCFAs on cells of the gastrointestinal tract, as well as immune cells (e.g., eosinophils and neutrophils) and adipocytes [13]. To date, there are no human intervention trials that have assessed how dietary fibre—in particular soluble fibre—can impact airway inflammation. Airway inflammation can be assessed in subjects with asthma by collecting induced sputum (which enables quantification of inflammatory cells) and the use of molecular techniques to measure gene expression to examine mechanistic pathways [14]. Furthermore, sputum can be induced serially, in a safe manner to assess changes in inflammation [15]. 

We hypothesised that increased intake of soluble fibre would reduce airway inflammation in asthma via activation of free fatty acid receptors. Therefore, in this pilot study, we aimed to investigate the effects of an acute soluble fibre challenge on airway inflammation and free fatty acid receptor activity in asthma.

## 2. Materials and Methods

### 2.1. Subjects

Twenty-nine subjects with stable asthma, aged ≥18 years and with a body mass index (BMI) between 18 and 30 kg/m^2^ were recruited from the John Hunter Hospital Asthma Clinic, NSW, Australia, from existing study volunteer databases and by advertisement. A subset of data from these participants has previously been reported [16]. Asthma stability was confirmed, defined as no exacerbation, respiratory tract infection, or oral corticosteroids in the past four weeks. Asthma control was assessed using the seven-item Asthma Control Questionnaire (ACQ-7) [17]. Clinical asthma pattern (intermittent, mild, moderate, or severe persistent) was determined according to the Global Initiative for Asthma (GINA) guidelines [18]. Subjects were excluded from the study if they were current smokers or had any respiratory-related illness other than asthma on presentation. This trial was conducted at the Hunter Medical Research Institute, Newcastle, Australia according to the guidelines laid down in the Declaration of Helsinki, and all procedures involving human subjects were approved by the Hunter New England Human Research Ethics Committee (HREC) (06/10/25/5.03, 11/06/15/4.02) and registered with the University of Newcastle HREC. Written informed consent was obtained from all subjects. The trial was registered with the Australian and New Zealand Clinical Trials Registry (number ACTRN12607000236493) prior to the study commencing. 

### 2.2. Study Design

All subjects fasted for 12 h, withheld short acting β_2_-agonist medications (for 12 h) and long acting β_2_-agonist medications (for 24 h), and underwent exhaled nitric oxide (eNO, Ecomedics CLD 88sp, Ecomedics, Duernten, Switzerland), spirometry, and hypertonic saline challenge with combined sputum induction, and then consumed the study meal (soluble fibre *n* = 17, control *n* = 12) within 15 min. Four hours after meal consumption, eNO, spirometry, and sputum induction were repeated. The soluble fibre meal consisted of commercial probiotic yoghurt (Vaalia, low fat, 175 g), containing 806 kJ, the soluble fibre inulin (3.5 g), and the probiotics *Lactobacillus acidophilus* strain LA5, *Bifidobacterium lactis* strain Bb12, and *Lactobacillus rhamnosus* strain GG, each at a concentration of ≥10^8^ colony forming units. The control meal consisted of an isocaloric meal of 200 g plain mashed potato. 

### 2.3. Hypertonic Saline Challenge

Baseline spirometry (Minato Autospiro AS-600; Minato Medical Science, Osaka, Japan) was performed to measure lung function, and predicted values for forced expiratory volume in one second (FEV_1_) and forced vital capacity (FVC) were calculated using NHANES (National Health and Nutrition Examination Survey) III data, which accounts for gender, age, and height [19]. Using predicted values for FEV_1_ and FVC allows for interpretation of actual lung function values (L) as a percent of expected values developed from reference data in healthy populations [20]. Combined bronchial provocation and sputum induction with nebulized (ULTRA-NEBTM ultrasonic nebulizer, Model 2000; DeVilbiss, Tipton, West Midlands, United Kingdom) hypertonic saline (4.5%) were performed as previously described [21]. If FEV_1_ dropped below 15% of baseline, subjects were considered to have airway hyperresponsiveness (AHR). The response of all subjects to hypertonic saline was also described by the dose response slope (DRS, the percent decline in FEV_1_ per mL hypertonic saline). 

### 2.4. Sputum Processing

Sputum was selected from saliva [21,22], dispersed with dithiothreitol, and a total cell count of leukocytes and viability were performed. Cytospins were prepared, stained (May-Grunwald Geimsa), and a differential cell count obtained from 400 non-squamous cells. The remaining solution was centrifuged (400× *g*, 10 min, 4 °C) and the cell-free supernatant was aspirated and stored at −80 °C. For GPR41 and GPR43 gene expression analysis, 100 µL of selected sputum was added to Buffer RLT (Qiagen, Hilden, Germany) and stored at −80 °C until RNA extraction. 

### 2.5. Sputum Supernatant Measurements

The concentration of interleukin 8 (IL-8) was determined by Enzyme-linked Immunosorbent Assay (ELISA) using R&D Systems Human CXCL8/IL-8 DuoSet Ancillary Reagent ELISA kit (R&D Systems, Minneapolis, MN, USA). IL-8 has been previously validated for assessment in induced sputum [23,24]. 

### 2.6. RNA Extraction and cDNA Synthesis

Total RNA was extracted from sputum samples stored in Buffer RLT using the RNeasy Mini Kit (Qiagen) as per kit instructions. Sputum RNA was quantitated using the Quant-iT RiboGreen RNA Assay Kit (Invitrogen, Eugene, OR, USA), whereby fluorescence was measured by FLUOstar OPTIMA spectrometer (BMG LabTech, Ortenberg, Germany). Genomic DNA was removed via treatment with DNase I (Life Technologies, Scoresby, Australia) and cDNA was generated via reverse transcription from 200ng of RNA using the High Capacity cDNA Reverse Transcription Kit, as per kit instructions (Applied Biosystems, Forster City, CA, USA).

### 2.7. mRNA Quantification by Real-Time qRT-PCR

Taqman gene expression assays for GPR41 and GPR43 were purchased as proprietary pre-optimised reagents (Applied Biosystems) and combined with cDNA and Taqman master mix in duplicate singleplex quantitative real-time polymerase chain reactions (qRT-PCR) (ABI 7500 Real time PCR system). 18S ribosomal RNA was utilised as the housekeeping reference gene. Relative fold change in gene expression after the soluble fibre and control meal challenge was calculated using 2^−ΔΔ*C*t^ relative to the housekeeping gene 18S rRNA (ΔCt) and the baseline (ΔΔCt).

### 2.8. Statistical Analysis

Data were analysed with STATA 11 (StataCorp, College Station, TX, USA) and reported as mean ± standard error of mean (SEM) for parametric and median (interquartile range, IQR) for nonparametric data. Data was tested for normality using the D’Agostino–Pearson omnibus normality test. To compare baseline characteristics between groups, normally distributed variables were analysed using Students *t*-tests, and non-parametric variables were analysed using the Wilcoxon rank sum test. Categorical variables were analysed by Fisher’s exact test. Within-group changes were compared using Wilcoxon matched-pairs signed rank test, and between group changes were analysed using the Wilcoxon rank sum test. Significance was accepted if *p* < 0.05. 

## 3. Results

### 3.1. Baseline Characteristics

Twenty-nine subjects with stable asthma were included in the study, allocated to the soluble fibre (*n* = 17) or control group (*n* = 12). The clinical characteristics at baseline were similar between the two groups (Table 1). 

### 3.2. Airway Inflammatory Markers

**Figure 1:**
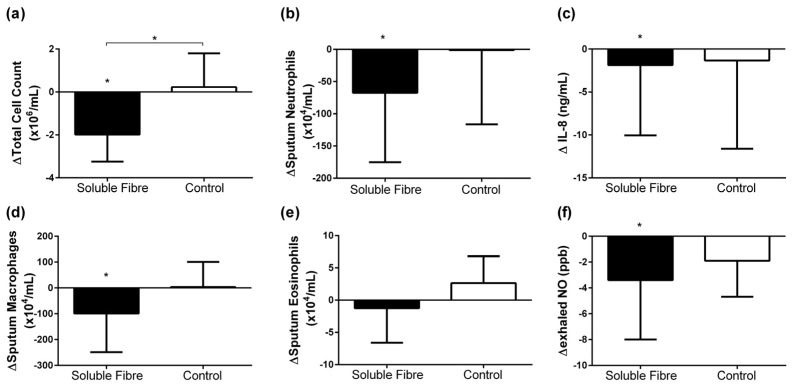
Change in airway inflammatory markers: (**a**) sputum total cell count; (**b**) sputum neutrophils; (**c**) sputum interleukin 8 (IL-8); (**d**) sputum macrophages; (**e**) sputum eosinophils; (**f**) exhaled nitric oxide 4 h following soluble fibre challenge (soluble fibre group) or control meal challenge (control group). Data expressed as median (IQR) and analyzed by Wilcoxon matched pairs signed-rank for within group and Wilcoxon rank-sum for between group comparisons. * *p* < 0.05.

There was no difference in airway inflammation at baseline between groups; however, the response to the soluble fibre and control meals was different (Table 2, Figure 1). Following the control meal, there were no changes in airway inflammation. Within the soluble fibre group, there was a significant decrease in sputum total cell count which was significantly different to the control group (Figure 1a). Sputum neutrophils (Figure 1b), macrophages (Figure 1d), lymphocytes, sputum IL-8 (Figure 1c), and eNO (Figure 1f) also decreased significantly in the soluble fibre group at 4 h compared to baseline, though these changes were not significantly different between the control and intervention groups. 

### 3.3. GPR41 and GPR43 Sputum Gene Expression

**Figure 2:**
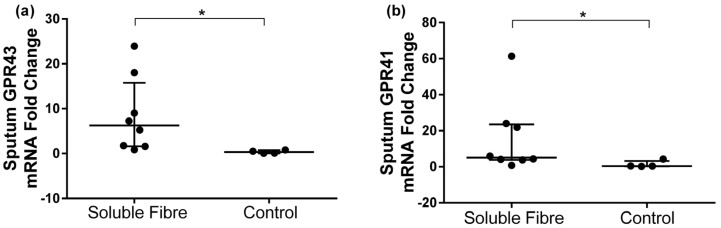
Change in sputum (**a**) GPR43 and (**b**) GPR41 gene expression 4 h following soluble fibre challenge (soluble fibre group *n* = 8) or control meal challenge (control group *n* = 4). Data expressed as median (IQR) and analyzed by Wilcoxon rank-sum test.* *p* < 0.05.

GPR41 and GPR43 gene expression fold change in sputum was compared between the two groups after the soluble fibre and the control meals. Both GPR43 and GPR41 expression were significantly upregulated following the soluble fibre meal compared to the control meal (Table 3, Figure 2).

### 3.4. Lung Function

**Figure 3:**
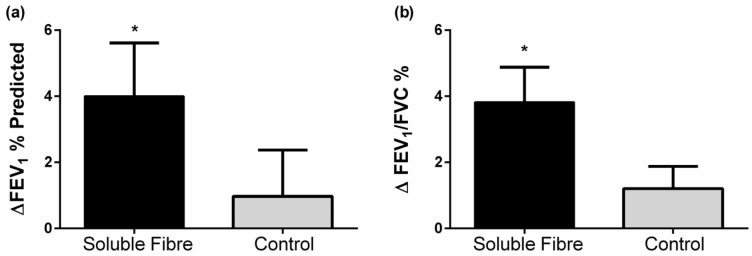
Change in lung function (**a**) FEV_1_% predicted and (**b**) FEV_1_/FVC % 4 h following soluble fibre challenge (soluble fibre group) or control meal challenge (control group). Data expressed as mean ± SEMs and analyzed by two-sample *t*-test. * *p* < 0.05.

At baseline, forced vital capacity (FVC % predicted) was lower in the control group, but both groups were within normal range (80–120) of percentage predicted FVC [25]. FEV_1_ and FEV_1_/FVC improved 4 h following the soluble fibre meal, while no changes in lung function were seen following the control meal (Table 4, Figure 3). There were no significant differences in lung function changes between groups.

## 4. Discussion

To gain a better understanding of the effects of soluble fibre on airway inflammation in people with asthma, this pilot study examined changes in airway inflammatory biomarkers at 4 h versus baseline (0 h), following a soluble fibre or control meal. We observed significant reductions in airway inflammation four hours after the consumption of a single dose of soluble fibre (3.5 g inulin). This was characterised by decreases in sputum total cell count, neutrophils, macrophages, lymphocytes, sputum IL-8 and eNO. When compared to the control subjects, we also found significantly upregulated GPR41 and GPR43 sputum cell gene expression in the soluble fibre group. We also observed an improvement in lung function (FEV_1_ and FEV_1_/FVC) in the soluble fibre group.

Several studies in the general population have reported an inverse relationship between dietary fibre intake and systemic inflammation [26,27]. We have previously extended these observations by demonstrating that dietary fibre is also inversely associated with eosinophilic airway inflammation in asthma [12]. The current study suggests that a key mechanism driving the anti-inflammatory actions of soluble fibre is the activation of free fatty acid receptors. The soluble fibre inulin is partially fermented by commensal bacteria in the colon, providing the substrate for production of physiologically active by-products, including the SCFAs: acetate, propionate, and butyrate. Our observations suggest that fermentation of inulin leads to activation of GPR41 and GPR43 in immune cells in the airways, which results in a reduction in airway inflammation. While we did not observe a reduction in airway eosinophils in the current study, we did observe a decrease in eNO, which is a marker of eosinophilic inflammation [28].

Our observations support findings from animal models, which have shown that GPR43/41 stimulation by SCFAs is necessary for the resolution of airway inflammation. In an allergic airways model, GPR43-deficient mice showed more severe inflammation, with increased inflammatory cell numbers in the lung lining fluid and higher levels of eosinophil peroxidase activity and inflammatory cells in lung tissue [29]. In another study in mice with allergic airways disease [30], a fibre-rich diet changed the composition of the gut microbiota by increasing proportions of the Bacteroidaceae and Bifidobacteriaceae families, which are potent fermenters of soluble fibre into SCFA. Increased circulating SCFA levels were observed and airway inflammation was attenuated, with dendritic cells having an impaired ability to activate Th2 effector cells in the lung [30]. Consequently, in mice fed either a high fibre diet or directly administered with acetate or propionate in their drinking water, airway inflammation could not be sustained; after an allergen challenge, cellular infiltration (eosinophils), IL-4, IL-5, IL-13, and IL-17A levels were reduced in the lungs and AHR improved. The effects were dependent on GPR41, but not GPR43. This highlights the importance of measuring both free fatty acid receptors in order to understand their anti-inflammatory actions.

This is the first study to examine the effects of SCFAs on inflammation in human airways. The majority of previous studies in humans that have looked at the role of SCFAs have examined gut inflammation, predominantly ulcerative colitis where sodium butyrate is administered either orally (using capsules with slow release coating to release butyrate into the colon), or through enemas [10]. We are interested in the effects of SCFAs on inflammation in peripheral tissues, in particular the lungs [31]. Certainly, it has previously been shown that soluble fibre can affect inflammation in the circulation. In a randomized, double-blind, placebo-controlled crossover study on inflammation and gut microbiota following soluble fibre supplementation, improvements in the composition and metabolic activity of gut microbiota—such as increased production of butyrate—was seen after two and four weeks [32]. In addition, significant reductions in circulating pro-inflammatory mediators such as tumour necrosis factor-alpha (TNF-α), IL-6, and IL-8 were observed [32]. We sought to extend our investigations to the airways, and have shown that a single dose of soluble fibre can modulate airway inflammation. This was associated with an improvement in lung function, and hence appears to be clinically important. 

The single meal challenge design that we employed mimics many previous studies that have shown that a single meal high in carbohydrates and/or fat causes postprandial systemic inflammation [33]. We have also previously shown that a single high fat mixed meal increases airway inflammation within 4 h [16]. This is biologically plausible, as it is known that at 4 h after the consumption of inulin, the SCFAs acetate, propionate, and butyrate are all elevated compared to baseline in plasma [34]. Nonetheless, a clinical trial of longer duration is warranted to determine what clinical effects would occur with chronic supplementation. 

As the importance of SCFAs in human health is being increasingly recognized, further research is required to assess changes that occur in circulating SCFA levels and inflammation following the intake of various types of soluble fibres. In addition, research is also needed to determine the optimal delivery form for increasing circulating SCFAs. In animal studies, SCFAs are often administered directly through the drinking water of animals, however oral delivery of SCFA is not optimal for humans, as the majority of acetate delivered orally is oxidized, with plasma levels only remaining elevated for 60 min [35]. Hence, supplementation with soluble fibre (as we have done in this study) is a useful strategy to increase circulating SCFA levels [34]. 

From our study, we are unable to determine whether SCFA reach the lungs, and this is an important area for future research. Previous animal studies have shown that SCFA are not detectable in lung tissue following soluble fibre supplementation [30]. Hence, it appears likely that effects of SCFA on immune function initially occur systemically. Supporting this hypothesis, it has been reported that mice treated with propionate have altered haematopoiesis in bone marrow, characterised by changes in the types of dendritic cell (DC) precursors generated, which have greater phagocytic ability but reduced ability to promote Th2 responses once in the lungs [30]. 

A limitation of this study is the small sample size; however, this was adequate for us to demonstrate, for the first time, that soluble fibre can reduce airway inflammation in people with asthma. Another potential limitation is the short duration of the study, as the soluble fibre was delivered as a single meal. Nonetheless, the chronic effects of soluble fibre on airway inflammation in asthma are likely to be even greater, as in addition to activation of free fatty acid receptors, gut microbial changes will also be induced, which further enhance SCFA production [36]. Hence, long-term studies of soluble fibre supplementation are warranted.

## 5. Conclusions

In summary, we have shown that a single dose of soluble fibre was able to significantly reduce airway inflammation in stable asthma. The long-term benefits of increasing soluble fibre intake are likely to have a greater impact on inflammation, as soluble fibre also enhances the growth of SCFA-producing gut bacteria, which will further enhance the production of beneficial SCFAs. Supplementing with a prebiotic such as inulin has the potential to be widely accepted and adopted, and could potentially reduce the amount of inhaled glucocorticoids required for treatment. As such, this approach provides a less costly strategy for managing asthma, as well as lowering the risk of asthma patients experiencing adverse side effects due to pharmacological treatment. 

## Figures and Tables

**Table 1:** Baseline clinical characteristics.

Clinical Characteristic	Soluble Fibre Group (*n* = 17)	Control Group (*n* = 12)	p *
Gender Male *n* (%)	7 (41)	6 (50)	0.716
Female *n* (%)	10 (59)	6 (50)	
Age (years)	42.1 ± 3.4	40.4 ±4.6	0.770
BMI (kg/m^2^)	24.9 ± 0.7	26.6 ± 0.7	0.099
Ex-smokers *n* (%)	6 (35)	3 (25)	0.694
ACQ-7 (units)	0.7 ± 0.1	0.7 ± 0.2	0.941
Atopy *n* (%)	14 (82)	8 (67)	0.403
AHR *n* (%)	10 (59]	5 (41.7)	0.462
DRS (%∆FEV_1_/mL), med [IQR]	2.2 [0.4, 6.4]	0.6 [0.2, 2.4]	0.109
ICS dose ^†^ (μg/day), med [IQR]	1000 [400, 1000]	1000 [563, 1000]	0.679

Data are means ± SEM unless otherwise stated. BMI: body mass index; ACQ: 7-item asthma control questionnaire; AHR: airway hyperresponsiveness; DRS: dose–response slope (% fall in forced expiratory volume in one second (FEV_1_)/mL saline); ICS: inhaled corticosteroids. * Difference between soluble fibre and control groups. ^†^ Beclomethasone equivalents.

**Table 2:** Airway inflammation at baseline and change at 4 h following soluble fibre or control meal challenge ^^^.

Airway Inflammation	Soluble Fibre Group		*p* ^†^	Control Group		*p* ^†^	*p* *
	0 h	∆4 h		0 h	∆4 h		
Sputum (*n*)	16	14		10	9		
TCC (×10^6^/mL)	3.7 [2.2, 6.3]	−2.0 [−2.8, −1.1]	0.013	3.0 [1.4, 6.0]	0.2 [−0.8, 1.4]	0.833	0.037
Neutrophils (×10^4^/mL)	76.2 [54.6, 249.4]	−67.5 [−158.0, −6.4]	0.033	92.4 [7.2, 342.7]	−1.1 [−94.9, 105.1]	1.000	0.148
Eosinophils (×10^4^/mL)	3.3 [1.3, 16.0]	−1.2 [−5.8, 1.8]	0.490	2.4 [0.0, 4.5]	2.6 [−1.7, 6.7]	0.440	0.393
Macrophages (×10^4^/mL)	144.0 [93.0, 386.3]	−99.1 [−244.6, −12.2]	0.030	124.5 [104.5, 211.5]	3.4 [−47.6, 85.1]	0.779	0.117
Lymphocytes (×10^4^/mL)	1.6 [0.2, 7.1]	−1.4 [−3.9, −0.3]	0.002	4.1 [0.4, 6.7]	2.1 [−1.5, 3.9]	0.401	0.034
Sputum IL-8 (ng/mL)	5.7 [3.9, 9.7]	−1.9 [−7.6, −1.1]	0.005	3.7 [3.0, 22.6]	−1.3 [−10.1, 1.2]	0.208	0.539
Exhaled NO (ppb)	17.5 [14.5, 70.5]	−3.4 [−7.9, −0.6]	0.028	14.9 [11.0, 51.0]	−1.9 [−4.6, 1.0]	0.170	0.301

TCC: total cell count; NO: nitric oxide; ppb: parts per billion. ^^^ Data are nonparametric, presented as median [quartile 1, quartile 3], within-group changes analyzed by Wilcoxon matched pairs signed-rank test, and group comparison performed using Wilcoxon rank-sum test. ^†^ Difference in change from 0 to 4 h within group. * Difference in change from 0 to 4hrs between the soluble fibre and control group.

**Table 3:** Sputum mRNA gene expression at baseline, 4 h, and fold change at 4 h following soluble fibre or control meal challenge ^^^.

Gene	Soluble Fibre Group (*n* = 8)			*p* ^†^	Control Group (*n* = 4)			*p* ^†^	*p* *
	0 h	4 h	FC		0 h	4 h	FC		
GPR43	0.07 [0.05, 0.29]	0.79 [0.26, 2.01]	6.26 [1.66, 13.5]	0.124	1.53 [0.29, 3.64]	0.24 [0.17, 0.27]	0.31 [0.07, 0.67]	0.068	0.007
GPR41	0.07 [0.04, 0.35]	1.70 [0.14, 5.18]	5.15 [3.96, 22.9]	0.161	1.27 [0.80, 1.42]	0.58 [0.41, 1.22]	0.41 [0.31, 2.37]	0.715	0.027

GPR: G protein-coupled receptor; FC: fold change. ^^^ Data are nonparametric, presented as median [quartile 1, quartile 3], within-group changes analyzed by Wilcoxon matched pairs signed-rank test and group comparison performed using Wilcoxon rank-sum test. ^†^ Difference in change from 0 to 4 h within group. * Difference in fold change between the soluble fibre and control group.

**Table 4:** Lung function at baseline and change at 4 h following soluble fibre or control meal challenge.

Lung Function	Soluble Fibre Group		*p* ^†^	Control Group		*p* ^†^	*p* *
	0 h	∆4 h		0 h	∆4 h		
(*n*)	17	17		12	12		
FEV_1_ (L) ^^^	2.7 [2.2, 3.6]	0.1 [0.0, 0.2]	0.022	2.9 [2.0, 3.5]	0.1 [−0.1, 0.2]	0.347	0.341
FEV_1_ (% predicted) ^#^	82.4 ± 4.4	4.0 ± 1.6	0.024	77.7 ± 6.8	1.0 ± 1.4	0.506	0.190
FVC (L) ^^^	4.1 [3.4, 4.7]	0.0 [−0.1, 0.1]	0.331	4.0 [3.5, 4.9]	0.01 [−0.04, 0.1]	0.503	0.595
FVC (% predicted) ^#^	98.9 ± 3.4 ^§^	1.6 ± 1.2	0.193	86.9 ± 4.5	−0.2 ± 1.0	0.859	0.285
FEV_1_/FVC (%) ^#^	69.2 ± 2.5	3.8 ± 1.1	0.002	71.9 ± 4.6	1.2 ± 0.7	0.101	0.070

FEV_1_: forced expiratory volume in 1 s; FVC: forced vital capacity. ^^^ Data are nonparametric, presented as median [quartile 1, quartile 3], within-group changes analyzed by Wilcoxon matched pairs signed-rank test and group comparison performed using Wilcoxon rank-sum test. ^#^ Data are normally distributed and presented as means ± SEMs, within group changes analyzed by paired *t*-test and group comparison performed using two-sample *t*-test. ^§^ *p* < 0.05 versus control group at baseline. ^†^ Difference in change from 0 to 4 h within group. * Difference in change from 0 to 4 h between the soluble fibre and control group.
